# Characterizing Neuronal Derived Extracellular Vesicles Using Single Molecular Array Technology

**DOI:** 10.1002/alz70861_108376

**Published:** 2025-12-23

**Authors:** Jennifer Pollock, Julia Miller, Connor Yew, Everett Gutterman‐Johns, Anubhav Tripathi

**Affiliations:** ^1^ Brown University, Providence, RI USA

## Abstract

**Background:**

Extracellular vesicles (EVs) were isolated from plasma using multiple different methods and their protein cargo was analyzed to evaluate how isolation methods effect downstream analysis.

**Method:**

EVs were isolated from normal plasma (n = 5) using immunoprecipitation, size exclusion chromatography (SEC), and ultracentrifugation. Immunoprecipitation was performed with three different neuron specific surface markers (NCAM, L1CAM, and ATP1A3). The total yield of amyloid‐beta (1‐40), amyloid‐beta (1‐42), GFAP, and NfL for each method was measured using a single molecular array and normalized against the total number of EVs isolated.

**Result:**

There was no significant difference in yield between EVs isolated using SEC and immunoprecipitation. There was no significant difference in yield between NCAM and ATP1A3. L1CAM immunoprecipitation yield significantly more total protein than NCAM, ATP1A3, and CD9. GFAP was determined to be localized to the surface of EVs in plasma. Amyloid‐beta (1‐40), amyloid‐beta (1‐42), GFAP, and NfL were all determined to be highly associated with neuronal EVs.

**Conclusion:**

Despite recent publications questioning the use of L1CAM as a surface marker for nEVs, it was determined that L1CAM immunoprecipitation yielded more protein than other neuronal EV markers. It was also observed that SEC and EV preconcentration did not effect downstream protein analysis when immunoprecipitation is performed.

